# Acute on Chronic Bilateral Subdural Hematoma in a Woman with a Remote History of Previous Subdural Hematoma Managed by Trephination: A Case Report

**DOI:** 10.31729/jnma.5122

**Published:** 2020-08-31

**Authors:** Mitesh Karn, Sapana Yonghang

**Affiliations:** 1 Gandaki Medical College Teaching Hospital and Research Center, Pokhara, Nepal

**Keywords:** *hematoma*, *subdural*, *trephination*

## Abstract

Bilateral chronic subdural hematoma are not that common. It may be recurrent and rarely superimposed by acute bleed leading to rapid progression and poor clinical outcomes. We report the case of a seventy six years old lady with a history of traumatic subdural hematoma evacuated by trephination twenty years back, presenting at our hospital with a history of persistent headache and acute onset of several episodes of vomiting. A non-contrast head CT revealed bilateral chronic subdural hematoma with acute on chronic bleed on one side. Trephination was done initially unilaterally, but the symptoms persisted and bilateral trephination was performed. The patient developed bilateral pneumocephalus and chest infection post-surgery. Bilateral, recurrent subdural hematoma with acute superimposition of bleed is a rare entity that presents with signs of increased intracranial pressure as opposed to unilateral SDH. A single burr hole trephination can be an effective intervention in these cases.

## INTRODUCTION

Chronic subdural hematoma (CSDH) is a common neurosurgical condition. Although unilateral CSDH occurs in most patients, bilateral CSDH is relatively rare. Rapid escalation of bilateral CSDH has been documented, including the risk of recurrence.^1,2^

Bilateral, recurrent CSDH with superimposition of acute bleed is a rare condition. We hereby report a case of bilateral CSDH with acute bleed unilaterally in a 76-years old lady with a history of previous traumatic SDH and an ominous surgical history.

## CASE REPORT

A seventy-six years old lady presented to our Emergency Department with complaints of progressive worsening of headache eleven days in duration and acute onset of several episodes of vomiting since the past two days. She was referred from another center where she was treated symptomatically with no relief of either pain or vomiting. She reported no weakness or numbness of upper and lower limbs. There was no history of fever, abnormal body movements and loss of consciousness. She reported decreased appetite and alteration of sleep habit. She did not give any history of recent trauma or falls. She however gave a history of head surgery 20 years back for a subdural hematoma that she had due to a fall and a leg fracture 7 years ago which was fixed with plates. An interesting revelation was that the patient had remained unconscious for three months following her previous surgery for SDH evacuation.

On examination, she was ill-looking. Her vitals were normal. Cardiovascular, respiratory and abdominal examination were normal with a blood pressure of 140/80 mm Hg , pulse rate of 80 beats per minute, a respiratory rate of 20 breaths per minute and oxygen saturation (Sp02) of 98%. She was afebrile, conscious with a Glasgow Coma Scale of 15 and normal neurologic exam, including that of cranial nerves. Tone, power and reflexes were normal in both upper as well as lower limbs. Sensations to all four limbs were also preserved. Palpation of spine revealed no tenderness or steps.

**Figure 1.**
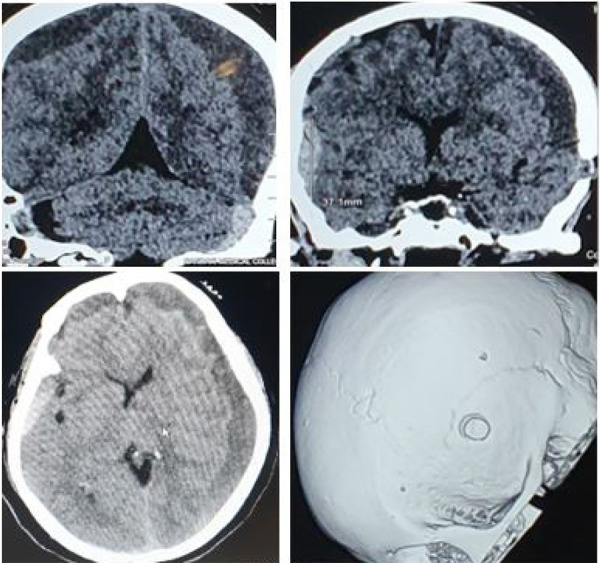
Initial non-contrast head CT showing bilateral CSDH with acute bleed on one side and a hole from previous trephination.

Initial laboratory investigations including random blood glucose, hematological profile including that of clotting, liver and renal function tests and other basic tests were normal. A non-contrast CT scan of the head was ordered in the ER which revealed a small hyperdense area surrounded by hypodense areas indicating acute on chronic, right subdural hematoma with a small acute bleed around the temporal lobe and a chronic bleed around the temporoparietal region. The CT also showed a massive hypodense area indicating a large subdural hematoma on the left temporoparietal region, with a very slight midline shift to the right, to go along with a previous burr hole mark on the left temporal bone (Figure 1).

**Figure 2.**
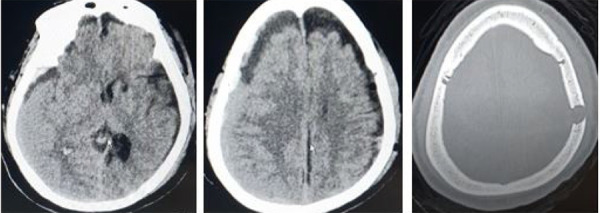
Postoperative follow up head CT showing expansion of unilateral hematoma and slight midline shift.

A neurosurgical referral was made. She was monitored intensively and treated conservatively with no improvement of her symptoms. Surgery was then planned. A left parietal burr-hole craniostomy was done under local anaesthesia, the following day with aspiration of “machinery-oil” blood under pressure. The headache resolved. The next day after the surgery, the patient complained of headache on the side opposite to which the procedure was performed. The patient was closely monitored in the neurosurgical Intensive Care Unit. After the pain persisted for a day more, a repeat head CT was ordered. It revealed isodense to slightly hyperdense areas in the right temporo-parietal region with a slight midline shift to the left (Figure 2).

**Figure 3.**
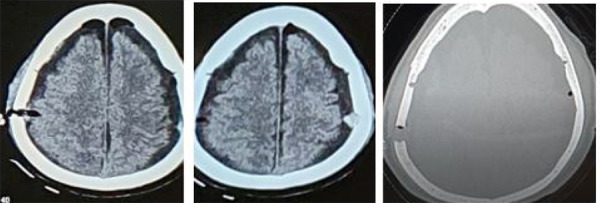
Follow up head CT after second surgery showing resolution of hematoma.

A right parietal burr-hole craniostomy was performed under local anaesthesia which led to aspiration of dark brown blood under pressure. A repeat CT of the head showed resolution of the hematoma (Figure 3).

**Figure 4.**
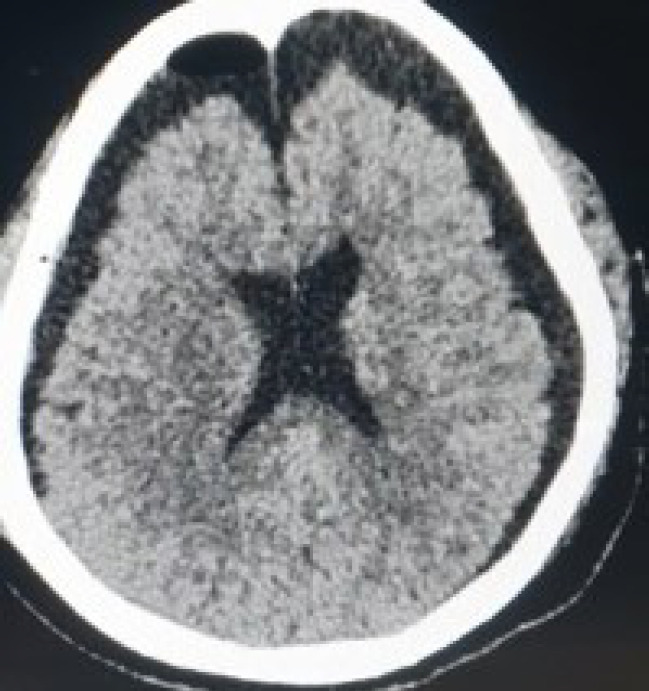
Resolution of pneumocephalus after aggressive management.

Three days after the second procedure, the patient complained of frontal headache and nausea. The patient was febrile as well. Repeat CT showed a large bi-frontal pneumocephalus (Figure 4).

**Figure 5.**
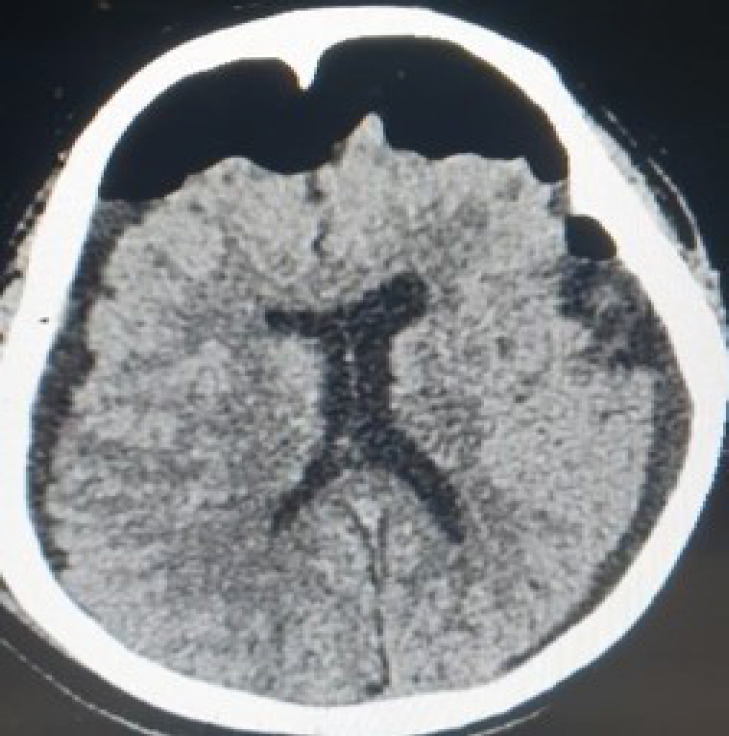
Bifrontal pneumocephalus complicating the surgery.

A chest radiograph showed bilateral infiltrates. These problems were managed medically with high flow oxygen therapy and intravenous antibiotics and the patient recovered eventually (Figure 5). She was discharged after 13 days of in-hospital stay.

## DISCUSSION

Subdural hematoma is defined by the accumulation of blood between the dura and the arachnoid layers of the meninges. It usually occurs due to the rupture of bridging veins, resulting in collection of subdural blood. Subdural hematomas can be acute, subacute or chronic. Acute subdural hematoma is commonly associated with traumatic, high energy impact and usually presents within twenty four hours of the inciting event. In contrast, chronic subdural hematomas usually are associated with mild or insignificant impact and become symptomatic more than two weeks after the inciting event. Bilateral CSDH have a reported incidence varying from 16% to 20%.^3,4^

Most of the cases of subdural hematoma are due to rupture of the bridging veins but about 20-30% cases are supposed to be due to arterial rupture.^5^ There are many identified risk factors of subdural hematoma, including cerebral atrophy due to ageing and in chronic alcoholics, anticoagulation, coagulopathy, decreased CSF pressure, aneurysmal rupture and arteriovenous malformation, neurosurgical procedures and infection.^6,7^

The clinical presentation of subdural hematomas vary greatly. Subdural hematoma is often referred as “great imitator” as it can mimic diseases such as stroke, dementia and parkinson's.^8^ Acute SDH results in coma in about half of the cases and in about 12 to 38 percent of patients have a transient interval that is followed by progressive neurologic decline to coma.^9^ Chronic SDH usually present with insidious onset of headaches, light-headedness, cognitive impairment, apathy, somnolence. Focal neurologic deficits may be ipsilateral or contralateral to the side of the lesion and the symptoms may be transient or fluctuating. Bilateral CSDH are reported to show signs of increased ICP such as headache but less likely to show symptoms associated with brain shift such as hemiparesis and are associated with rapid progression and poor clinical outcomes.

Surgical evacuation of CSDH is recommended if it is symptomatic and associated with signs of increased ICP. The surgical evacuation of symptomatic SDH can be performed by different techniques, including burr hole trephination, craniotomy, and decompressive craniectomy.^11^ While burr-hole craniostomy continues to be the most commonly used technique for the treatment of CSDH, successful treatment proves a challenging dilemma due to a 10-20% recurrence rate.^12^ Our patient had a history of a two month unconsciousness following her first burr hole trephination which we expected to be due to some anaesthetic used during that procedure. That is why more invasive methods were not recommended to her and trephinations were performed under local anaesthesia.

Conclusively, we can say that CSDH can present in the elderly without any inciting event or with minimal impact. Bilateral CSDH usually present with signs of increased intracranial pressure in contrast to signs of focal deficit and brain shifts seen with unilateral SDH. Unilateral evacuation may lead to expansion of the contralateral hematoma, requiring bilateral surgery. In the elderly population with an ominous surgical history, single burr hole trephination under local anaesthesia can be a better and safer alternative to more invasive procedures.
